# Multimodal Image Analysis in Alzheimer’s Disease via Statistical Modelling of Non-local Intensity Correlations

**DOI:** 10.1038/srep22161

**Published:** 2016-04-11

**Authors:** Marco Lorenzi, Ivor J. Simpson, Alex F. Mendelson, Sjoerd B. Vos, M. Jorge Cardoso, Marc Modat, Jonathan M. Schott, Sebastien Ourselin

**Affiliations:** 1 Translational Imaging Group, CMIC, UCL, London, UK; 2 MRI Unit, Epilepsy Society, Chalfont St Peter, UK; 3 Dementia Research Centre, UCL Institute of Neurology, Queen Square, London, UK

## Abstract

The joint analysis of brain atrophy measured with magnetic resonance imaging (MRI) and hypometabolism measured with positron emission tomography with fluorodeoxyglucose (FDG-PET) is of primary importance in developing models of pathological changes in Alzheimer’s disease (AD). Most of the current multimodal analyses in AD assume a *local* (spatially overlapping) relationship between MR and FDG-PET intensities. However, it is well known that atrophy and hypometabolism are prominent in different anatomical areas. The aim of this work is to describe the relationship between atrophy and hypometabolism by means of a data-driven statistical model of non-overlapping intensity correlations. For this purpose, FDG-PET and MRI signals are jointly analyzed through a computationally tractable formulation of partial least squares regression (PLSR). The PLSR model is estimated and validated on a large clinical cohort of 1049 individuals from the ADNI dataset. Results show that the proposed non-local analysis outperforms classical local approaches in terms of predictive accuracy while providing a plausible description of disease dynamics: early AD is characterised by non-overlapping temporal atrophy and temporo-parietal hypometabolism, while the later disease stages show overlapping brain atrophy and hypometabolism spread in temporal, parietal and cortical areas.

The multimodal analysis of anatomical and physiological images is of primary importance in developing comprehensive models of biological processes and pathologies, and increasing the statistical power of current imaging biomarkers. Already, both brain atrophy, measured in magnetic resonance images (MRIs), and hypometabolism, quantified by positron emission tomography with fluorodeoxyglucose radiotracers (FDG-PET), are among the primary diagnostic biomarkers of Alzheimer’s disease (AD). The information provided by these two imaging modalities is correlated, since hypometabolism and neuronal loss are interdependent biological phenomena. However, at the present moment, a joint model of the hypometabolism-atrophy relationship in AD has not been developed, and current hypotheses on their interaction are mostly based on the quantification of grey matter volume and FDG uptake at the regional level.

In recent years, voxel-wise approaches to multimodal analysis in AD have been proposed^1^. In particular, image synthesis techniques based on machine learning have been used to synthesise FDG-PET images from MRIs of AD patients for diagnostic purposes^2^,^3^. The majority of these approaches are based on the local modelling of the relationship between MR and FDG-PET signals, either by considering the voxels independently, or through neighborhoods (patches) defined around voxels. However, it is well known that the link between morphology and function in the brain is not purely local^4^. For this reason, local methods may provide only a limited description of the link between structure and function in AD.

Several techniques have been proposed for modelling non-overlapping signal correlations in the field of functional MRI analysis. For instance, both independent component analysis (ICA) or partial least square (PLS) approaches have been successfully applied to the joint analysis of functional activation in the brain and covariates drawn from genetic, clinical, or imaging data^5^,^6^,^7^. In the context of correlation modelling in multimodal imaging analysis, multivariate techniques such as PLS have the appealing characteristic that they do not rely on any hypothesis about the spatial overlap between voxels’ signals. They are thus able to model relationships between non-adjacent voxels. Unlike purely local correlation model approaches (for example, those based on voxel-wise correspondencies or on patch-based search windows), these methods optimise the *latent components* describing the *global* correlation of the images treated as multidimensional arrays. This enables them to model the potentially significant interactions between voxels located in completely different areas of a single image, or between voxels in images of different modalities.

Several multivariate approaches have previously been applied to the multimodal analysis of imaging data in neurodegenerative diseases^8^. Notable approaches include parallel ICA, which has been used to analyze the relationships between brain amyloid deposition and either atrophy or hypometabolism^9^,^10^, and canonical correlation analysis, which has been used to study the correlation between structural connectivity and brain atrophy^11^. Though PLS itself has been previously applied in the joint analysis of brain metabolism and atrophy^12^,^13^, past analyses have been limited to relatively small clinical samples and have focused on solely the first latent component. The use of higher-order components may aid in the discovery of more complex correlation structures, though it brings with it greater challenges related to stability and replicability.

The aim of this work is to investigate the spatial relationship between brain atrophy and hypometabolism in a large clinical cohort of the ADNI dataset, by means of a data-driven PLS statistical model of non-overlapping intensity correlations. This is achieved by applying a computationally tractable formulation of PLS regression (PLSR) to the joint analysis of non-local intensity correlations in FDG-PET and T1 weighted MR images. Unlike previous studies, in this work we extend PLSR to the analysis of the high-order latent components, and we introduce a thorough cross-validation scheme in order to identify the reproducible and biologically relevant latent components of joint correlation.

The performance of the PLSR model is compared to a common non-parametric approach for multimodal image analysis based on local intensity similarities. The experimental validation shows that the proposed PLSR approach outperforms the local reference analysis in terms of predictive accuracy while providing an interpretable, reproducible and biologically plausible description of the spatial relationship between atrophy and hypometabolism in AD.

## Local vs Non-local Correlation Models of Imaging Data

In this section, we introduce the computational models used to compare non-local and local assumptions of multimodal intensity correlation. The respective models are a computationally tractable application of PLSR to non-local intensity correlation in image data^14^ and a non-parametric model based on local patch similarities^15^,^16^.

### Computationally Tractable PLSR in Imaging Data

In the following, let 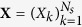 and 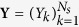 be the matrices of predictor and predicted image modalities respectively, where 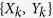 is the multimodal image pair sampled at the same voxel grid, of subject *k*. We assume that **X** and **Y** correspond to T1-MR and FDG-PET respectively. The size of **X** and **Y** is 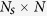, where 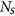 is the number of individuals, *N* is the number of image voxels, and the images are represented by row vectors.

The partial least squares (PLS) approach is based on the decomposition of the observations through a projection onto *m*-dimensional latent spaces defined by the basis vectors 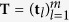 and 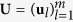 such that 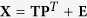, and 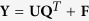, where **P** and **Q** are the associated coefficients, and **E** and **F** are matrices of residuals. In particular, PLS aims to maximise the covariance of the projections in the latent space: 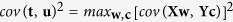, where **w** and **c** are unitary basis vectors of the latent space. Several formulations of PLS have been proposed in different research contexts^17^,^18^,^19^,^20^,^21^, and it can be shown that the solution of PLS can be obtained from the principal vectors of the singular value decomposition (SVD) of the covariance matrix 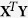
18. PLS can be iteratively computed as follows:

Let 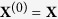, 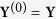. Iterate over the index 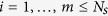:

1. *SVD step.* Compute the principal eigen-vectors 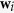 and 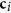 of the SVD decomposition of the matrix 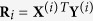.
2. Compute the latent vectors 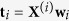, and 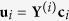, and corresponding coefficients 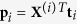.
3. *Deflation step.* Decorrelate the data from the principal directions: 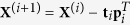, and 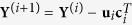.

We note that the matrix 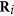 is usually very large (voxels × voxels), and its SVD decomposition is generally computationally infeasible. However, the SVD step can still be efficiently computed from the eigen-value problem associated with the matrix 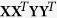, which is usually of much smaller dimension 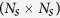. This approach has been proposed previously in^14^, which focused on the analysis of within-modality non-local intensity correlations in neuroimages. In particular, this efficient optimization scheme was used to model either group-wise patterns of cortical thickness from MRI, or functional connectivity networks measured in fMRI. In this work, we apply this computational approach in the context of multimodal analysis of brain images.

PLSR builds upon the above formulation of PLS by assuming a linear relationship between the vectors **t** and **u**, i.e. 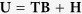, where **B** is a latent linear mapping and **H** is the residual matrix. The PLS model can thus be rewritten as 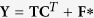, where 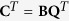, and 
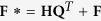
 is the residual error. It can be shown that the solution of the PLSR is 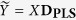 where the regression coefficient 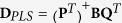, and 
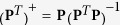
 is the right Moore-Penrose pseudoinverse of 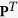
20.

### PLSR model of the non-overlapping spatial correlations in multimodal images

PLSR has a number of appealing features that can be exploited in the context of high-dimensional modelling of multimodal images. First, the basis of spatial eigen-components, 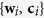, provides a *parsimonious* and low-dimensional representation of the multimodal correlation pattern, and can be used for exploratory analysis and modelling purposes. Second, the loadings 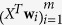 are a low-dimensional representation of the individual anatomy that can be used to address quantitative analysis problems, such as group-wise comparisons or classification. Finally, PLSR defines a transfer function linking the two modalities and, given an individual image *X*, provides a prediction of the associated target modality *Y* according to the model of non-local signal correlation estimated in the data 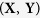.

### Relationship between PLSR and principal component regression

Another intuitive approach to multimodal correlation modelling in image data is principal component regression (PCR). This consists of an initial PCA step applied separately to the predicted and predictor variables, and a subsequent regression step to model the correlations between the resulting low dimensional representations. Both PCR and PLSR yield a predictive model using low dimensional latent space, but they differ in how this is driven; while PLSR aims to find a subspace that directly maximises the covariance between the predicted and predictor variables, PCR selects a latent space that maximises the variability within each variable set separately. For this reason, it may include components that are not useful in characterising the relationship between variable sets which may degrade predictive performance. We therefore prefer PLSR. The interested reader can find an experimental illustration of the differences between PLSR and PCR when applied to the problem analysed in this study in Appendix A.

### Local Models of Intensity Correlations Based on Patch Similarities

Patch-based methods are becoming a popular approach for the estimation of non-linear signal correspondences between different modalities. They have found several successful applications in medical image analysis, such as multi-modal image registration^15^, or in FDG-PET synthesis^2^. This approach is based on the assumption that, given an individual image *X*, the intensities of the target modality 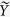 can be inferred from the local intensity correspondences observed in a database of atlas pairs 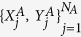
22. The between-modality voxel-to-voxel mapping 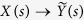 is usually not explicitly modelled in a parametric way, but is indirectly inferred from the local intensities of the images 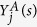 corresponding to the atlases 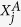 most correlated with *X*. The local correlation model presented here is the same as the one proposed in state-of-art approaches in the context of FDG-PET image synthesis^16^. All subjects images are aligned to the target using non-linear registration, and the intensity at a given location is estimated using the most similar patch in the database as determined using the local intensity information (the *L*^2^ metric). The chosen patch size was of 5 voxels.

## Analysis of Brain Hypometabolism and Atrophy in Alzheimer’s Disease

### Study Participants

Data used in the preparation of this article were obtained from the Alzheimer’s Disease Neuroimaging Initiative (ADNI) database (adni.loni.usc.edu, Date of Access: 04/2013). The ADNI was launched in 2003 as a public-private partnership, led by Principal Investigator Michael W. Weiner, MD. The primary goal of ADNI has been to test whether serial magnetic resonance imaging, positron emission tomography, other biological markers, and clinical and neuropsychological assessment can be combined to measure the progression of mild cognitive impairment and early Alzheimer’s disease.

Patients were selected when both MR and FDG-PET images were available at the baseline timepoint. The resulting study cohort consisted of 1049 subjects: 274 healthy controls, 168 patients affected by AD, and 607 patients with mild cognitive impairment (MCI). Of the latter, 154 subsequently converted to AD during the time of the study. Clinical and socio-demographical information are reported in Table 1.

### Image Processing

FDG-PET images were obtained at the standardised resolution of 8 mm FWHM, and normalised using the mean intensity in the cerebellar grey matter. T1-weighted MR images at both 1.5 and 3.0 Tesla were included to increase the size of the available sample. A sample specific group-wise space was defined for our analysis using iterative non-rigid registration and averaging the grey matter segmented from the MR images. Registration was performed using the freely available nifty-reg package^23^, and grey matter and FDG-PET images were resampled to the group-wise space. The resampled grey matter images were modulated by the corresponding Jacobian determinant of the template-to-subject transformation, and subsequently spatially filtered at the point spread function of the PET images and downsampled. Thanks to the modulation and to the downsampling operation, the resulting anatomical areas of apparent ventricular and CSF expansion are associated to smaller voxel-wise intensity values due to the scaling by the Jacobian determinant values.

### Statistical Analysis

#### Model Estimation and Comparison

**Figure 1:**
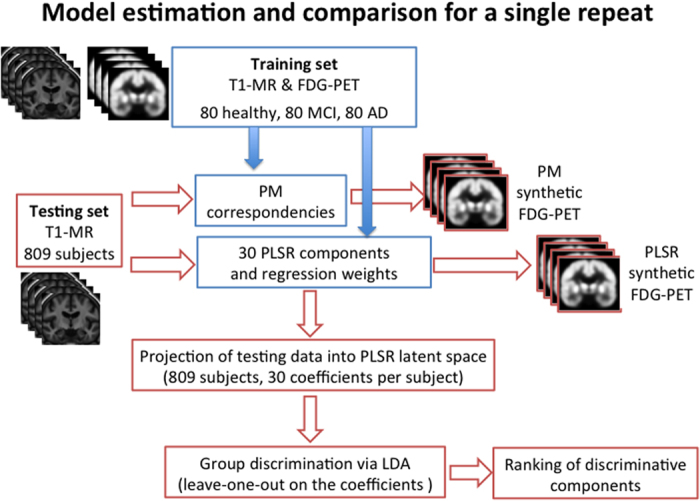
Flowchart of the cross-validation scheme of a single repeat in the proposed experimental setting.

The goodness of fit of PLSR and local approaches was assessed by cross-validation. The training data was composed of 80 healthy controls, 80 MCI, and 80 AD patients, randomly chosen from the study cohort. The data were respectively used to 1) estimate the PLSR latent components and regression coefficients, and 2) as an atlas database for the local patch-based method (PM). The PLSR model was computed by estimating 30 latent components. The model could be ideally computed by estimating the 239 latent basis-components corresponding to 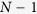 training data samples. The number of estimated PLSR components was limited to 30 for practical reasons because, as shown in the next experimental section, the stability and reproducibility of the high-order components is generally very low, and they usually provide very little contribution to the model performance. The resulting multimodal correlation models were validated on the remaining subjects. The experiment was repeated 10 times with different training sets to ensure the generalisation of the results. Due to the non-parametric nature of PM, we also compared the PM trained with a leave-one-out scheme (1048 training samples per test) in order to use of the largest amount of training data. The predictive accuracy was measured by the absolute difference between predicted and observed FDG-PET in temporal, posterior and parietal cortices, and by comparing the average predicted FDG-PET regional values to the SUVR values independently reported in the ADNI dataset. Figure 1 illustrates the flowchart of the cross-validation scheme of a single repeat adopted in the proposed experimental setting.

#### Reproducibility and Biological Plausibility of PLSR model

We investigated the biological plausibility of the non-local correlation pattern of the PLSR model. For this purpose, a linear discriminant analysis (LDA) was performed on the coefficients of the latent space associated with the testing subjects, in order to identify the mostly discriminative PLS components through leave-one-out. The discriminative accuracy of the PLSR model was quantified by computing the area under the receiver operating characteristic (ROC) curve associated to the LDA classification result.

The interpretation of the PLSR modes of correlation is usually challenging, since some of the obtained components (especially at the high order) tend to be noisy and not necessarily related to meaningful anatomical interpretation (an illustrative example of the set of the first 10 components estimated by PLSR in a single repeat is shown in Appendix B). For this purpose, in order to address this important issue of robustness, we measured the reproducibility of the most discriminative components across the 10 repeats. We were interested primarily in the rate of reproduction of the individual components, rather than in their relative ordering. To this end, the discriminative power of each component was quantified by the absolute value of the associated LDA weight, and the resulting 5 mostly discriminative components of each experiment were matched to those of the other repeats. Components were matched when the absolute value of the correlation between them exceeded 0.5. When multiple matches were possible, the strongest was chosen. The resulting labelling establishes the reproducibility of the discriminative components across repeats, and does not necessarily reflect the order of the eigen-components estimated in each PLS run.

## Results

### Model comparison

**Figure 2:**
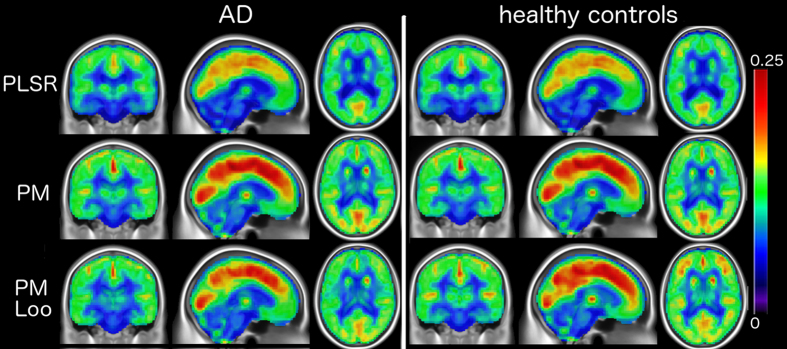
Mean absolute prediction error of PLSR and PM (240 training samples, and leave-one-out -Loo-). PLSR provides higher predictive accuracy than the local patch based (PM) approach. Results are similar when considering the MCI group (not shown).

Figure 2 shows the average pattern of absolute differences of the predictions in AD and healthy controls with both methods. We notice that PLSR generally provides a better fit, while the local patch based method leads to larger estimation errors in parietal and temporal areas. We note that increasing the training sample size slightly improves the PM, especially in the temporal regions. The average regional absolute error between predicted images and real ones was systematically higher for PM as compared to PLSR, and significantly different for 8 out of 10 repeats (p < 0.01, paired t-test). The PLSR prediction also provided significantly better agreement with the ADNI measurements than PM (Table 2A). This is reflected by the significantly higher effect size associated with the average measures for the PLSR approach, indicating a better separation between clinical groups (Table 2B).

### Biological Plausibility of the PLSR model

**Figure 3:**
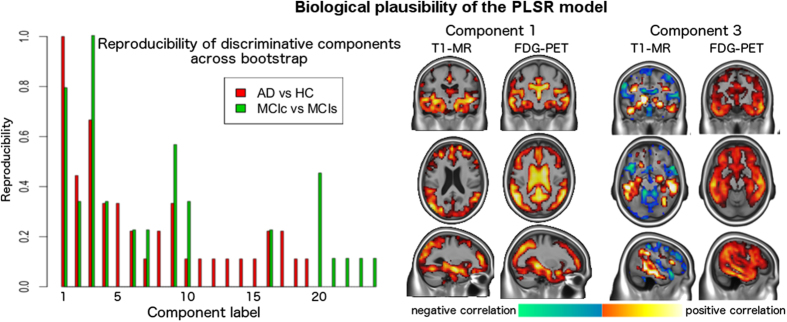
Left: reproducibility of the discriminative components. When comparing AD and controls, component 1 was the only one 100% reproducible and discriminative across repeats. The same consideration applies to component 3 when comparing stable and converting MCI. Right: component 1 describes the relationship between atrophy and FDG-PET uptake spread in temporal, parietal and posterior regions. We also note the partial volume effect in the ventricles for the FDG component. Component 3 shows the *non-overlapping* spatial inverse relationship between increased expansion of the CSF (ventricles and brain sulci), and joint increased temporal atrophy and cortical hypometabolism.

Figure 3, left, shows the reproducibility results for the PLSR components across repeats. The components are ordered according to the order of output in the PLSR results obtained in the first cross-validation repeat. The reproducibility results are instead quantified by the green and red bars, which indicates the number of times that a component was among the most discriminative across repeats. We note that only few of the mostly discriminative PLSR components are highly reproducible across repeats. In particular, the first eigen-component estimated in each PLSR repeat is the most discriminative when comparing AD vs healthy controls, while the third one is most discriminative when comparing both MCI stable and converters, and healthy controls and MCI converters. Both components were 100% reproducible across repeats. We note that since the figure is relative to the first cross validation repeat, the components estimated during the other repeats that did not find any match in the reproducibility analysis are omitted in the figure. However, apart from the reported components 1 and 3, no other reproducible component associated to the other repeats was observed in the analysis.

**Figure 4:**
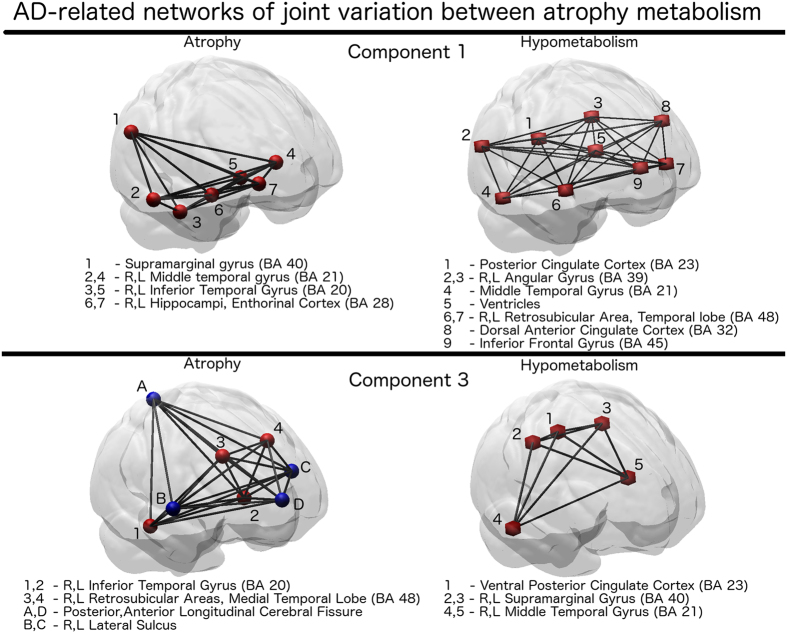
PLSR networks of joint relationship between atrophy and hypometabolism derived from Component 1 and Component 3. Red hubs indicate regions of joint within- and between-modality correlation. Blue hubs indicate anticorrelated regions (CSF expansion measured in T1-MR). BA = Broadmann anatomical areas.

These components are shown on the right hand side of Fig. 3, and the associated correlation network is shown in Fig. 4. A 3D rendering of the correlation networks is shown at the following url: https://www.dropbox.com/s/orsf3nt6hq2kp38/supplementary_animations1.mov?dl=0 (12/10/2015). The networks were obtained by thresholding components 1 and 3, and by subsequently applying a morphological opening operation in order to identify a consistent set of clusters of maximal PLSR weights.

On one hand, we note that component 1 describes the relationship between atrophy and FDG-PET uptake spread in temporal, parietal and posterior regions. In particular, it shows the partial volume effect due to ventricular expansion in AD, that is already observable in the raw data, and that leads to the very large variation of the FDG-PET signal in the ventricles in subjects with pronounced global brain atrophy. On the other hand, component 3 shows the non-overlapping inverse correlation pattern between increased expansion of the CSF, and joint increased temporal atrophy and cortical hypometabolism.

Finally, the average area under the ROC curve for the classification tasks across the different folds was 0.87 (0.83, 0.91–95% c.i.) for the comparison of AD vs healthy controls, and 0.75 (0.73, 0.76–95% c.i.) for the comparison between stable and converting MCI. This result primarily confirms the ability of the proposed PLSR to model biologically relevant features, and is in line with the classification performance based on T1-MR information previously reported in the literature on the ADNI dataset^24^,^25^,^26^.

## Conclusions

We have investigated the problem of multimodal analysis of biomedical images in AD, by comparing two different modelling hypothesis based on state-of-art techniques, PLSR and patch-based local correlation, to promote non-local correlation analysis approaches with respect to localized ones in describing multimodal correlation patterns in AD. Our study introduces and validates the use of PLSR in the context of multimodal modelling in AD by showing that PLSR bases and coefficients can be estimated in very large datasets of volumetric images through a computationally tractable approach to the eigen-decomposition.

### Non-local vs local multimodal modelling in AD

Our results show that the proposed non-local approach outperforms classical PM-based multimodal local correlation models in terms of modelling accuracy and predictive power. The ensemble of the reported results proves the ability of the proposed PLSR in capturing biologically relevant features, and in generalising to unseen structural imaging data of T1-MR scans.

Even though the presented study does not provide a theoretical proof of the superiority of non-local methods, our results show that T1-MR and FDG-PET present reproducible and consistent patterns of correlations between non-overlapping anatomical areas. This study thus shows that realistic multimodal models of neurodegeneration necessarily need to account for the non-local relations intimately related to the neurobiological aspects of the disease.

### Plausibility of the PLSR model

PLSR provides a parsimonious description of the global biological variability, represented by the low-dimensional latent subspace parameterisation. For this reason, the interpretation and statistical analysis of PLSR is more straightforward than that of the usually complex models provided by non-parametric local approaches^2^.

Our analysis revealed that in the sequential stages of the pathology (cognitively normal −>MCIc −>AD) we can consistently identify two reproducible components of correlation between atrophy and hypometabolism. Our results are therefore supportive of the existence of different patterns of atrophy and hypometabolism which differentially characterise the different stages of the disease, and thus are informative of the dynamics of the pathology.

The correlation networks highlighted in this study are supported by known biological dynamics between atrophy and hypometabolism in dementia^4^: although hypometabolism and atrophy are typically locally correlated, i.e. areas with neuronal loss (atrophy) show by definition reduced or absent metabolism, hypometabolism may be seen in areas not obviously or typically affected by atrophy, as exemplified by focal dementia syndromes, such as posterior cortical atrophy.

This work shows that T1 weighted MRI and FDG-PET in AD are highly correlated and share important patterns of common non-overlapping spatial relationship. The proposed method could be used in the future to identify and decorrelate the common inter-modality variation from biomedical images for the identification of more specific image based biomarkers.

## Additional Information

**How to cite this article**: Lorenzi, M. *et al*. Multimodal Image Analysis in Alzheimer,s Disease via Statistical Modelling of Non-local Intensity Correlations. *Sci. Rep.* **6**, 22161; doi: 10.1038/srep22161 (2016).

## Supplementary Material

Supplementary Information

## Figures and Tables

**Table 1:** Baseline socio-demographical and clinical information of the cohort of this study.

	**healthy**	**MCI stable**	**MCI conv**	**AD**
N	274	453	154	168
age (years)	74.1 (5.98)	72.16 (7.55)	73.21 (7.37)	75.66 (7.66)
sex (% females)	48	42	41	42
education (years)	16.22 (2.77)	16.01 (2.73)	16.03 (2.67)	15.1 (3.08)
MMSE	28.98 (1.21)	28.11 (1.66)	27.02 (1.75)	23.05 (2.1)

The entries for age, education and MMSE indicate group-wise mean and standard deviation in parenthesis. MMSE: mini mental state exam.

**Table 2:** 

**A. Correlation wrt ADNI SUVR**			**B. Effect size**		
	**PLSR**	**PM**		**PLSR**	**PM**
Whole cohort*	0.31 (0.25,0.38)	0.21 (0.14, 0.27)	AD vs HC *	1.07	0.79
AD and HC*	0.33 (0.22, 0.43)	0.23 (0.12, 0.34)	MCIc vs MCIs	0.53	0.37
MCIc and MCIs*	0.30 (0.22, 0.38)	0.20 (0.12, 0.28)	MCIc vs HC*	0.67	0.46

A. Correlation (mean, 95% confidence interval) between predicted average regional FDG-PET and the corresponding SUVR values reported in ADNI. B. Effect size between the measures obtained with PLSR and with PM. HC: healthy controls, MCIc: MCI converted to AD, MCIs: MCI stable. (* for significant differences, p < 0.05, paired t-test).
