# Supplementary material for: Multimodal Image Analysis in Alzheimer’s Disease via Statistical Modelling of Non-local Intensity Correlations
Source: Sci Rep. 2016 Apr 11;6:22161. doi: 10.1038/srep22161 (PMC4827392; doi:10.1038/srep22161)
Supplement: Supplementary Information [file srep22161-s1.pdf]

Multimodal Image Analysis in Alzheimer's Disease via Statistical Modelling of Non-local Intensity Correlations

Marco Lorenzi[1], Ivor J. Simpson[1], Alex F. Mendelson[1], Sjoerd B. Vos[1,2], M. Jorge Cardoso[1], Marc Modat[1], Jonathan M. Schott[3], Sebastien Ourselin[1], and [2]

[1]Translational Imaging Group, CMIC, UCL, London, UK

[2]MRI Unit, Epilepsy Society, Chalfont St Peter, UK

[3]Dementia Research Centre, UCL Institute of Neurology, Queen Square, London

Appendix

A Comparison with respect to Principal component regression

We replicated the analysis of section 3 by computing the first 30 principal eigen-components by PCA for T1-MR and FDG-PET data separately. The problem was efficiently addressed by using the numerical scheme adopted in our work for the estimation of the PLSR components. We then applied two

different kind of regression approaches, linear SVM and random forest (RF) (thus non-linear regression), to the data projected on the resulting T1-MR components, in order to predict the eiegen-components associated to FDG-PET, and to identify the mostly predictive ones of T1-MR.

In supplementary Figures 1 and 2, we show the feature weights associated by respectively SVM and RF to each T1-MR component. We can clearly see that although the regression models can quite clearly associate the first two components to the respective ones of FDG-PET, for the higher order components there is not an unique and clear association. For example, component 3 of T1-MR is associated to component 1 of FDG-PET. This is probably due to the well known high variability of the high order eigen-components in PCA, as well as generally in spectral methods. We conclude that in the context of the analysis proposed in our study, PLSR is indeed better suited in terms of clarity and robustness.

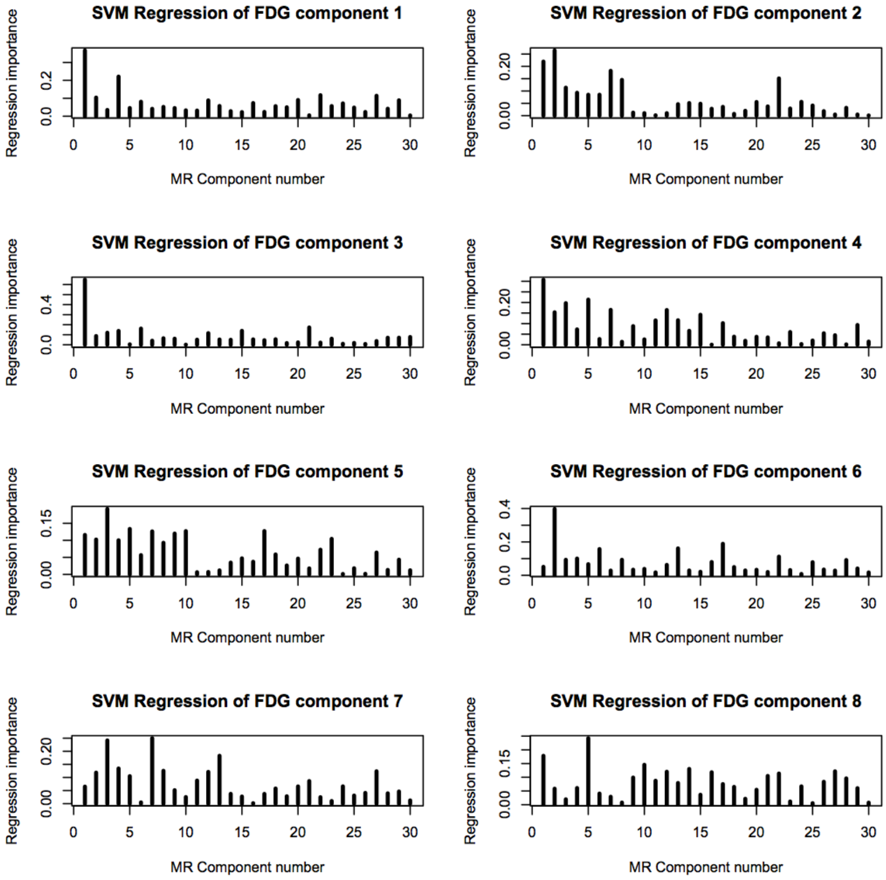

Figure 1: Feature importance for support vector regression in predicting the eigen-components associated to FDG-PET. The component 1 of T1-MR is strongly associated to component 1 of FDG-PET. However, the association for the higher order components is often unclear and not unique.

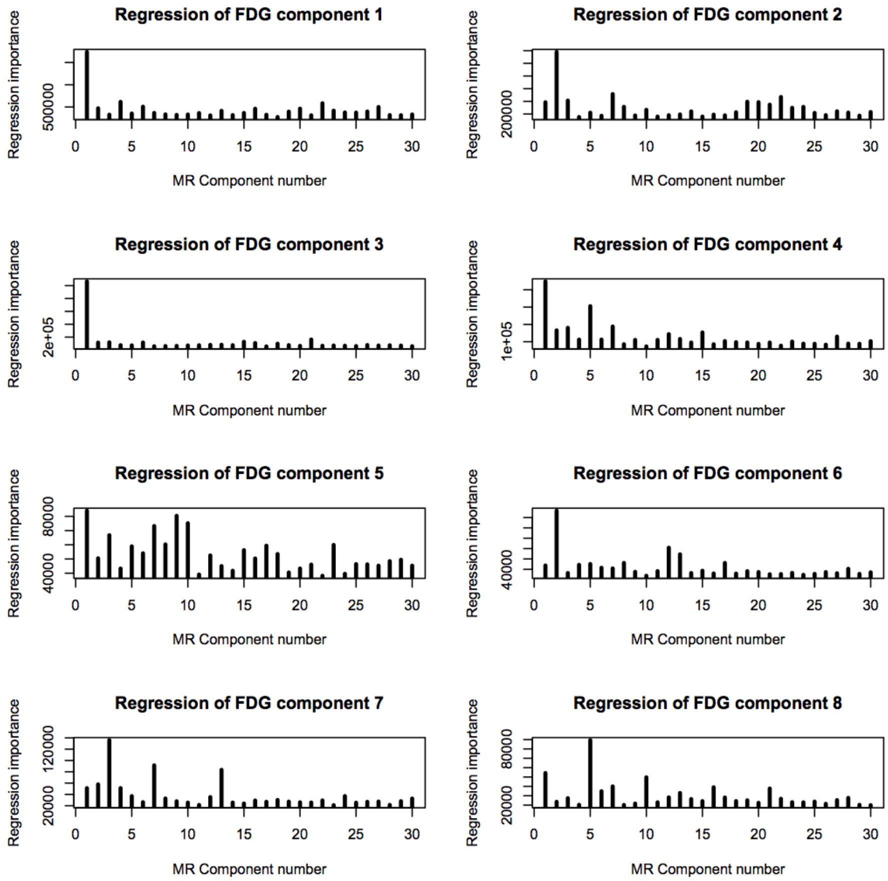

Figure 2: Feature importance for random forest regression in predicting the eigen-components associated to FDG-PET. The component 1 and 2 of T1-MR are strongly associated respectively to components 1 and 2 of FDG-PET. However, the association for the higher order components is often unclear and not unique.

B Illustration of PLSR components

In this section we show the first 10 components estimated by PLSR in a single repeat. The figures provides an insightful illustration of the challenge represented by the interpretation of the resulting modes of correlation. In fact, some of the obtained components (especially at the high order) tend to be noisy and not necessarily related to meaningful anatomical interpretation.
For this reason, the proposed cross-validation and analysis of consistency and biological plausibility is a fundamental step in order to provide reliable and stable results. Indeed, our validation results show that out of the several components provided by PLSR, only two of them (number 1 and 3) are i) reproducible across repeats, and ii) consistently related to variation associated to the pathology.

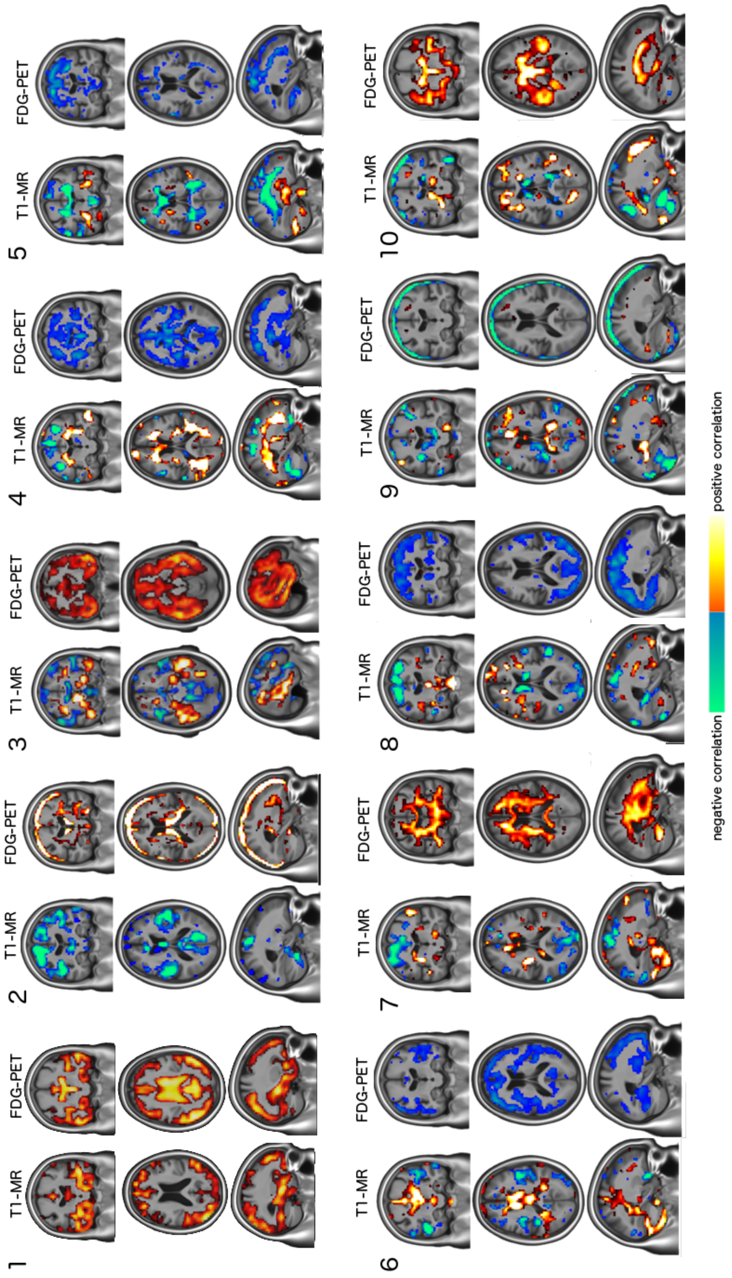

Figure 3: First 10 principal components of joint variation estimated by PLSR in a single repeat.
